# Capacity evaluation for general practitioners in Pudong new area of Shanghai: an empirical study

**DOI:** 10.1186/s12939-016-0484-8

**Published:** 2016-11-28

**Authors:** Ming Li, Zhiqun Shu, Xuan Huang, Zhaohui Du, Jun Wu, Qingshi Xia, Kun Liu, Jiquan Lou, Limei Jing

**Affiliations:** 1 Shanghai Pudong Institute for Health Development, Shanghai, China; 2 Pudong New Area Commission of Health and Family Planning, Shanghai, China; 3 Medical Institutions Administration Center of Pudong New Area, Shanghai, China

**Keywords:** General Practitioner, Capacity Evaluation, Formative Evaluation, Human Resource, Primary Health Care, Health Service, Empirical Study, Preliminary Study, Shanghai

## Abstract

**Background:**

Building highly qualified General Practitioners (GPs) is key to the development of primary health care. It’s therefore urgent to ensure the GPs’ quality service under the background of the new round of health care system reforms in China. A new model of GP qualification examination was originally implemented in Pudong New Area of Shanghai, China, which aimed to empirically evaluate the GPs’ capability in terms of clinical performance and social recognition. In the current study, an analysis was made of the first two years (2014–2015) of such theoretical and practical examinations on the GPs there with a view to getting a deep insight into the GP community so as to identify the barriers to such a form of GP qualification examination.

**Methods:**

The agency survey method was applied to the two-year database of the GP examinees, the formative research conducted to explore the key elements for developing the examination model. The data analysis was performed with SPSS for Windows (Version 19.0) to describe the GPs’ overall characteristics, and to make comparisons between different groups.

**Results:**

In 2015, the total number of GPs was 1264 in the area, in different districts of which, statistically significant differences were found in sex, age, professional title and employment span (*P* < 0.05). Such results were found to be similar to those in 2014. The examinees’ theoretical scores were statistically different (F = 7.76; *P* < 0.05), showing a sloping trend from the urban district to the suburban, to the rural and then to the farther rural, as indicated by LSD-*t* test (*P* < 0.05). From the theoretical examinations the scores were higher on the western medicine than on the traditional Chinese medicine (F = 22.11; *P* < 0.05).

**Conclusions:**

As suggested by the current study on the GPs’ qualification examination, which was pioneered in Pudong New Area of Shanghai, the construction of GP community was far from sufficient. It was a preliminary study and further studies are merited along the construction and development in terms of continuing medical education, performance appraisal and incentive mechanism.

## Background

In the Opinions of the Central Committee of the Communist Party of China and the State Council on Deepening the Health Care System Reforms in 2009 [1], to promote the construction of the four health systems of the public health service system, medical service system, medical insurance system and drug supply system was the most important goal issued, and the gross medical institutions as the base of the Chinese health service system has been playing an important role in public health. Admittedly, the construction of the primary health care is based on the development of the community health service (CHS) in rural China. At the CHS centers (CHSCs) the general practitioners (GPs) provide the local residents with basic health care. The issued guidelines on establishing the communities of GPs have advocated their importance in the national health strategy [2]. Dr. Michael Dixon, National Health Service Alliance chair, once said, “Numerous researches (e.g., from Barbara Starfield and WHO) have shown that a health service predicated on primary care delivers better mortality statistics, improved health, and is more cost effective” [3]. Thus, to build highly qualified GPs is critically important to the development of primary health care, and to promote the construction of GPs is to strengthen the primary healthcare system and improve the general health of Chinese populations.

At present, China encounters such healthcare problems as GPs’ insufficiency [4, 5], under-qualified personnel [6–11], uneven distribution [12, 13], and serious brain drain [14–16]. Thus the field studies and policy strategies focusing on in GPs’ human resource equity are of great importance to ensuring the quantity and quality of GPs in China. In March, 2010, the National Development and Reform Commission (NDRC), the National Health and Family Planning Commission (NHFPC), the State Commission Office of Public Sectors Reform (SCOPSR), the Ministry of Education (ME), the Ministry of Finance (MF), the Ministry of Human Resources and Social Security (MHRSS) jointly issued the Construction Planning of GP-Focused Primary Health Care Teams, which consisted of three major tasks of cultivation, employment and management to have built a community of 300,000 across the country by 2020 to address the requirements of primary health care [17]. Hereinto, an emphasis was placed on the establishment of ability-and-performance-oriented and social-recognition-focused mechanism of qualification evaluation targeted at the primary care personnel, because such an endeavor would help reduce inequity of health workforce in the provision of primary health care.

Over the world, more than 50 countries have health care systems based on the GP model [18]. Some of those countries such as UK, Germany and USA have developed advanced GP management system which integrates capacity evaluation with pre-occupation schooling and post-occupation training, performance appraisal and incentive mechanism throughout a GP’s entire career [19–23]. Different from those developed countries in the world, most countries are just at the beginning, trying to establish such a management system with its own characteristics. As an important part of medical practice in China, traditional Chinese medicine (TCM) plays a vital role in delivering primary health care. At the CHSC, the GPs who are engaged in the integration of TCM and western medicine (WM) can be expected to have routinely a holistic view and evidence-based skill in diagnosing and treating common diseases as well as in practicing rehabilitation and prevention. Technically, these types of GPs are certificated physicians of general medicine; therefore, they are equally treated as those counterparts of WM.

Previous investigations have presented multiple perspectives on GPs’ capacity evaluation [24–27]. In China, there have been few studies discussing GPs’ necessary ability elements [28] and the establishment of evaluation index system [7, 29]. Obviously, few studies have empirically evaluated GPs’ capacity in terms of ability and performance and recognition.

In 2014, a newly developed model of GP qualification examination, called the GP’s Theoretical and Practical Examination (GPTPE), was initiated in Pudong New Area of Shanghai, China, which was characterized by ability/performance-oriented evaluation so that the theoretical knowledge and practical capacity of the GPs could be evaluated at their working CHSC and the ways of alliance for other existing continuing medical education (CME) and clinical performance evaluation as well as GPs’ incentive mechanisms could be explored.

In the current study, we analyzed the first two-year data of Pudong-New-Area-based GPTPE (2014–2015) with a view to getting a deep insight into the GP community so as to identify barriers in particular to its further development, as empirical evidence for the national establishment of GP evaluation mechanism as well as for the promotion of equity in the human resource for the delivery of primary health care across the country.

## Methods

### Data sources

The first two-year data of GPTPE were derived from Pudong-New-Area-based Medical Institutions Administration Center (MIAC), which runs and manages GPTPE under the auspice of the Department of Medical Administration of Pudong New Area Commission of Health and Family Planning (CHFP). The training center of Weifang CHSC, exclusively designated as the examination place, was connected to the database. All the examinees were informed of the importance and necessity of GPTPE before they signed up. The managers of each CHSC could have their independent choice to take the examination or not. The scoring results would be linked with their annual performance appraisal.

The agency survey method was applied to exploring the general picture of Pudong-New-Area-based GPs based on the two-year database. The whole sample of the GPs’ self-administered information covered name, gender, age, working unit, employment span, practicing category and professional title.

And also the agency survey method was used to collect the examinees’ test scores, theoretical and practical, from 2014 to 2015 for evaluation. The theoretical test is taken within the computer paperless test system, with 100 questions selected at random and each representing one score, and the questions refer to the disciplines of general practice, internal medicine, surgery, gynecology, ophthalmology, otolaryngology, pediatrics and medical ethics. The practical test is taken by approximately 30% of the examinees selected randomly, with a maximum score of 100 for the operation on medical patient simulators.

The formative research served to explore the key elements for developing the new model of GPTPE [30, 31], which typically refer to such questions as who the target populations are; how the data are accurately evaluated; and what the conclusion is after the evaluation. A formative evaluation was made of the instantiation of GPTPE, which had been established on the basis of the mix-method research, through observation, statistics and interview, etc.

### Data analysis

The data analysis was performed with SPSS for Windows (Version 19.0), the threshold of statistical significance set at *P* < 0.05 (2-tailed). Based on the descriptive statistics on the GPs’ overall characteristics such as testing year, quantity, gender, age, regional distribution, practicing category, etc., comparisons were made among different groups using the F test for numerical variables and the *χ* ^2^ test for categorical variables.

## Results

### GPs in Pudong new area

As indicated by Table 1, Pudong-New-Area-based GPs numbered 1227 including the employees after retirement in 2014, and 1264 excluding employees after retirement in 2015. By 2015, 45 CHSCs had been in operation with 93 posts across the area. As defined by the degree of urbanization within the area, the GPs’ regional distribution could be administratively divided into 4 types: Type A as the urban area, Type B as the suburban, Type C as the rural and Type D as the farther rural. The different types of region were statistical different in sex, age, professional title, employment span (*P* < 0.05).

**Table 1:** Characteristics Data of GPs in Pudong New Area from 2014 to 2015 (% in Parentheses)

Characteristics	2014						2015					
	Type-A	Type-B	Type-C	Type-D	Total	*χ* ^2^/F	Type-A	Type-B	Type-C	Type-D	Total	*χ* ^2^/F
Sex												
male	134 (32.8)	78 (29.1)	104 (38.9)	117 (41.3)	433 (35.3)	11.74*	140 (33.2)	79 (28.5)	102 (36.8)	119 (41.3)	440 (34.81	11.20*
female	275 (67.2)	190 (70.9)	163 (61.1)	166 (58.7)	794 (64.7)		282 (66.8)	198 (71.5)	175 (63.2)	169 (58.7)	824 (65.2)	
Age												
~ 35	137 (33.5)	84 (31.4)	54 (20.2)	78 (27.5)	353 (28.8)	22.68*	142 (33.6)	79 (28.5)	50 (18.0)	70 (24.3)	341 (27.0)	25.36*
36 ~ 45	198 (48.4)	137 (51.1)	171 (64.1)	161 (56.9)	667 (54.3)		219 (51.9)	154 (55.6)	183 (66.1)	165 (57.3)	721 (57.1)	
46 ~ 55	53 (13.0)	37 (13.8)	30 (11.2)	37 (13.1)	157 (12.8)		53 (12.6)	40 (14.4)	41 (14.8)	46 (16.0)	180 (14.2)	
56~	21 (5.1)	10 (3.7)	12 (4.5)	7 (2.5)	50 (4.1)		8 (1.9)	4 (1.3)	3 (1.1)	7 (2.4)	22 (1.7)	
mean	38.9	38.9	39.94	39.24	39.2	F = 1.34	38.52	38.99	39.91	39.83	39.23	F = 3.69*
SD	8.05	7.08	6.98	6.23	7.22		6.84	6.3	6.18	6.27	6.47	
median	38	37	39	38	38		38	38	39	39	39	
minimum	25	26	25	26	25		26	27	26	27	26	
maximum	66	61	66	60	66		60	60	59	59	60	
Practical Category												
WM	323 (79.0)	224 (83.6)	225 (84.3)	241 (85.2)	1013 (82.6)	5.72	334 (79.2)	232 (83.7)	233 (84.1)	244 (84.7)	1043 (82.5)	5.08
TCM	86 (21.0)	44 (16.4)	42 (15.7)	42 (14.8)	214 (17.4)		88 (20.8)	45 (16.3)	44 (15.9)	44 (15.3)	221 (17.5)	
Professional Title												
junior	76 (18.6)	50 (18.7)	51 (19.1)	77 (27.2)	254 (20.7)	23.67*	26 (6.1)	45 (16.3)	63 (22.7)	82 (28.5)	216 (17.1)	80.24*
intermediate	306 (74.8)	195 (72.7)	209 (78.3)	199 (70.3)	909 (74.1)		367 (87.0)	207 (74.7)	204 (73.7)	200 (69.4)	978 (77.4)	
senior	27 (6.6)	23 (8.6)	7 (2.6)	7 (2.5)	64 (5.2)		29 (6.9)	25 (9.0)	10 (3.6)	6 (2.1)	70 (5.5)	
Employed Years												
~ 10 years	121 (29.6)	72 (26.9)	40 (15.0)	56 (19.8)	289 (23.5)	32.40*	119 (28.2)	59 (21.3)	42 (15.1)	50 (17.4)	270 (21.4)	34.92*
11 ~ 20 years	186 (45.5)	128 (47.8)	141 (52.8)	136 (48.1)	591 (48.5)		187 (44.3)	134 (48.4)	116 (41.9)	125 (43.4)	562 (44.5)	
21 ~ 30 years	65 (15. 9)	48 (17.9)	61 (22.8)	74 (26.1)	248 (20.2)		88 (20.9)	71 (25.6)	95 (34.3)	89 (30.9)	343 (27.1)	
31 ~ years	37 (9.0)	20 (7.4)	25 (9.4)	17 (6.0)	99 (8.1)		28 (6.6)	13 (4.7)	24 (8.7)	24 (8.3)	89 (7.0)	
mean	16.09	16.71	18.42	17.87	17.15	F = 4.87*	16.47	17.51	19.49	19.14	17.97	F = 10.82*
SD	9.48	8.65	8.09	7.73	8.66		8.27	7.8	7.72	7.8	8.03	
median	15	17	18	17	17		16	18	20	19	19	
minimum	1	1	1	1	1		2	1	2	1	1	
maximum	46	46	44	43	46		42	41	42	42	42	

*WM* western medicine, *TCM* traditional Chinese medicine, *SD* standard deviation

*P < 0.05 (2-tailed)

### GPTPE in 2014 and 2015

The GPs’ GPTPE scores of 2014 and 2015 were obtained from the theoretical and practical, respectively (Tables 2 and 3). A total number of 1189 and 1234 examinees took GPTPE in 2014 and 2015, respectively, except the managers of CHSCs who chose not to take it and those who had asked for maternity leave, sick leave or personal leave in advance. A random selection of 325 out of 1189 examinees (27.33%) and of 427 out of 1234 examinees (34.60%) was designated to take the practical test in 2014 and 2015, respectively.

**Table 2:** GPs’ Scores in Theoretical Exam from 2014 to 2015

Year	Category	Number	Mean	SD	95%CI	Minimum	Maximum	F	*P*
2014	CHSC								
	Type-A	393	57.42	10.57	(56.37 ~ 58.47)	29	90	13.86	0.00*
	Type-B	261	57.7	10.95	(56.37 ~ 59.04)	31	91		
	Type-C	260	55.83	10.68	(54.52 ~ 57.13)	23	90		
	Type-D	275	52.72	9.39	(51.60 ~ 53.83)	27	80		
	Sex								
	male	412	54.84	9.96	(53.88 ~ 55.81)	29	84	8.19	0.00*
	female	777	56.68	10.86	(55.92 ~ 57.45)	23	91		
	Age								
	~35	344	57.81	9.65	(56.78 ~ 58.83)	31	86	22.44	0.00*
	36 ~ 45	651	56.35	10.71	(55.52 ~ 57.17)	27	91		
	46 ~ 55	151	53.89	10.24	(52.25 ~ 55.54)	34	90		
	56~	43	44.91	9.64	(41.94 ~ 47.87)	23	64		
	Practical Category								
	WM	979	57.36	10.24	(56.72 ~ 58.00)	23	91	91.71	0.00*
	TCM	210	49.92	10.05	(48.56 ~ 51.29)	27	90		
	Professional Title								
	junior	247	54.44	10.81	(53.09 ~ 55.80)	27	84	6.90	0.00*
	intermediate	887	56.25	10.51	(55.55 ~ 56.94)	23	91		
	senior	55	60.00	9.83	(57.34 ~ 62.66)	39	89		
	Employed Years								
	~10 years	284	57.02	10.51	(55.79 ~ 58.25)	23	86	22.39	0.00*
	11 ~ 20 years	573	57.50	10.29	(56.65 ~ 58.34)	27	91		
	21 ~ 30 years	243	54.18	10.43	(52.86 ~ 55.50)	35	90		
	31 ~ years	89	48.69	9.50	(46.68 ~ 50.69)	29	73		
	Total	1189	56.05	10.59	(55.44 ~ 56.65)	23	91		
2015	CHSC								
	Type-A	412	55.34	8.11	(54.56 ~ 56.13)	33	76	7.76	0.00*
	Type-B	268	55.16	8.73	(54.11 ~ 56.21)	26	78		
	Type-C	273	53.56	8.51	(52.55 ~ 54.58)	34	82		
	Type-D	281	52.61	7.86	(51.69 ~ 53.53)	24	74		
	Sex								
	male	425	53.05	8.16	(52.27 ~ 53.83)	26	82	14.34	0.00*
	female	809	54.94	8.38	(54.36 ~ 55.51)	24	78		
	Age								
	~35	332	57.40	7.61	(56.58 ~ 58.22)	35	75	34.10	0.00*
	36 ~ 45	708	53.84	8.04	(53.24 ~ 54.43)	24	82		
	46 ~ 55	173	51.23	8.86	(49.90 ~ 52.56)	28	75		
	56~	21	45.48	7.10	(42.25 ~ 48.71)	33	60		
	Practical Category								
	WM	1013	54.80	8.51	(54.28 ~ 55.33)	24	82	22.11	0.00*
	TCM	221	51.91	7.13	(50.97 ~ 52.86)	34	75		
	Professional Title								
	junior	214	51.83	8.94	(50.63 ~ 53.04)	28	74	21.26	0.00*
	intermediate	959	54.52	8.11	(54.00 ~ 55.03)	24	82		
	senior	61	59.30	7.11	(57.47 ~ 61.12)	44	74		
	Employed Years								
	~10 years	263	56.65	7.66	(55.72 ~ 57.58)	35	74	31.49	0.00*
	11 ~ 20 years	550	55.28	7.71	(54.63 ~ 55.93)	34	82		
	21 ~ 30 years	336	52.24	8.63	(51.32 ~ 53.17)	24	78		
	31 ~ years	85	48.64	9.08	(46.68 ~ 50.59)	33	75		
	Total	1234	54.29	8.35	(53.82 ~ 54.75)	24	82		

*WM* western medicine, *TCM* traditional Chinese medicine, *SD* standard deviation, *CI* confidence interval

*P < 0.05 (2-tailed)

**Table 3:** GPs’ Scores in Practical Exam from 2014 to 2015

Category	2014						2015					
	Number	0 point	50 points	100 points	*χ* ^2^	*P*	Number	Mean	SD	95%CI	F	*P*
CHSC												
Type-A	113	13	48	52	3.7	0.72	147	76.73	15.324	(74.24 ~ 79.23)	2.04	0.11
Type-B	69	9	34	26			90	75.44	16.069	(72.08 ~ 78.81)		
Type-C	70	10	33	27			95	71.79	16.741	(68.38 ~ 75.20)		
Type-D	73	12	37	24			95	75	12.944	(72.36 ~ 77.64)		
Sex												
male	108	16	49	43	0.26	0.88	155	75.68	14.438	(73.39 ~ 77.97)	0.50	0.48
female	217	28	103	86			272	74.58	15.908	(72.68 ~ 76.48)		
Age												
~ 35	106	8	46	52	8.74	0.07	115	76.39	14.381	(73.73 ~ 79.05)	1.57	0.19
36 ~ 45	193	33	92	68			241	74.17	15.750	(72.17 ~ 76.17)		
46 ~ 55	26	3	14	9			65	76.38	14.510	(72.79 ~ 79.98)		
56~	—	—	—	—			6	65.00	24.900	(38.87 ~ 91.13)		
Practical Category												
WM	263	32	125	106	2.21	0.33	343	74.62	15.784	(72.94 ~ 76.30)	0.93	0.34
TCM	62	12	27	23			84	76.43	13.612	(73.47 ~ 79.38)		
Professional Title												
junior	67	6	32	29	1.92	0.75	69	74.71	15.948	(70.88 ~ 78.54)	2.74	0.07
intermediate	249	37	115	97			334	74.52	15.541	(72.85 ~ 76.19)		
senior	9	1	5	3			24	82.08	8.836	(78.35 ~ 85.81)		
Employed Years												
~ 10 years	82	5	37	40	9.47	0.15	95	74.21	16.443	(70.86 ~ 77.56)	0.78	0.50
11 ~ 20 years	168	26	75	67			182	76.29	14.195	(74.22 ~ 78.37)		
21 ~ 30 years	71	12	38	21			119	73.91	15.502	(71.09 ~ 76.72)		
31 ~ years	4	1	2	1			31	73.71	18.256	(67.01 ~ 80.41)		
Total	325	44	152	129			427	74.98	15.382	(73.51 ~ 76.44)		

*WM* western medicine, *TCM* traditional Chinese medicine, *SD* standard deviation, *CI* confidence interval

In different types of region, the theoretical scores of GPTPE were statistical different in 2014 (F = 13.86; *P* < 0.05), showing a sloping trend of Type A & B > Type C > Type D, as indicated by further LSD-*t* test (*P* < 0.05), while the practical scores of GPTPE showed no significant difference (*χ* ^2^ = 3.70; *P* > 0.05). In the theoretical GPTPE, the female scores were higher than the male ones (F = 8.19; *P* < 0.05), and a decline was observed there with an increase in age, as indicated by F test (F = 22.44; *P* < 0.05) and LSD-*t* test (*P* < 0.05). A trend of employment span was observed as follows: −10 & 11–20 years > 21–30 years > 31+ years, as indicated by F test (F = 22.39; *P* < 0.05) and LSD-*t* test (*P* < 0.05). As to the different practical categories, GPs of western medicine scored significantly higher in the theoretical GPTPE than the counterparts of TCM (F = 91.71; *P* < 0.05). Furthermore, the theoretical scores of GPTPE increased with the escalating professional title, as indicated by F test (F = 6.90; *P* < 0.05) and LSD-*t* test (*P* < 0.05), respectively.

Similar data were found in 2015; the theoretical scores of GPTPE were statistical different in different types of region (F = 7.76; *P* < 0.05), showing the same sloping trend as that in 2014, as indicated by LSD-*t* test (*P* < 0.05), and the GPs of WM scored higher there than the counterparts of TCM (F = 22.11; *P* < 0.05).

### Development of GPTPE Model

**Fig. 1:**
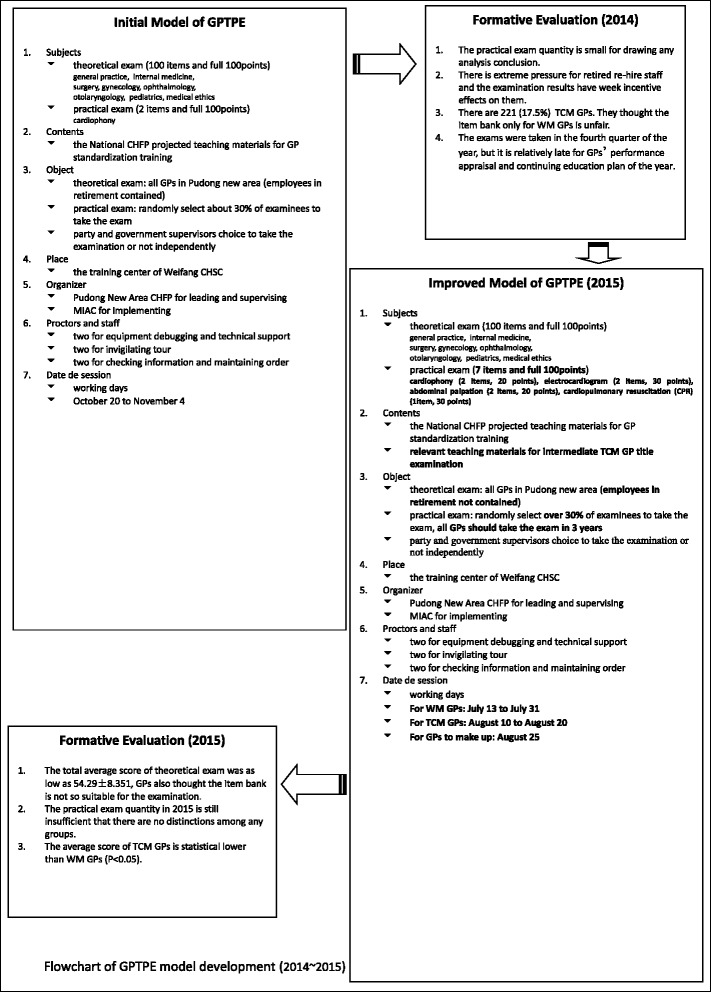
Flowchart of GPTPE model development (2014 ~ 2015)

On the basis of the initial model of GPTPE, the formative evaluation of the instantiation was performed from 2014, and after an analysis-remodification cycle, the model was improved in 2015 (Fig. 1).

In 2014, the practical GPTPE contained 2 items so that a possible score was 0, 50 or 100; therefore, it didn’t show any differences between groups. The post-retirement employees complained that it was of extreme pressure for them to take GPTPE and that the test results would have little incentive effect on them. A total number of 221 TCM examinees (17.5%) took GPTPE in 2014, who in reality composed an important part of primary health care at CHSC and reported that it was unfair because the test bank did not contain any well-targeted questions for them. In the seasonality of GPTPE, it was relatively late for the GPs to take the examination in the fourth quarter of the year as their performance appraisal and as part of their CME plan.

According to the empirical evidence of GPTPE in 2014, some improvement were made in some aspects of the model in 2015: 1) The practical GPTPE was expanded from 1 to 4 disciplines covering 7 items totally scored 100; 2) The test bank of theoretical GPTPE grew to contain 1040 questions, which were separately arranged for the examinees of WM and TCM; 3) The post-retirement employees did not have to take the examination; 4) The number of randomly selected examinees for the practical GPTPE was increased to be over 30% so that all GPs could take it in 3 years; and 5) In case of system failure or other temporary problems, a whole day was scheduled for the examinees to make up.

In 2015, a total number of 1234 out 1264 GPs (97.63%) took GPTPE, the rate higher than that in 2014 (1189/1227; 96.90%). The TCM examinees’ average score was 51.91 ± 7.13 in the theoretical GPTPE, which was higher than that (49.92 ± 10.05) in 2014 (t = 2.38; *P* < 0.05), while it was significantly lower than that (54.80 ± 8.51) of the WM ones (F = 22.11; *P* < 0.05). Additionally, in 2015 the total average score of the theoretical GPTPE was as low as 54.29 ± 8.35, which the examinees blamed for the unsuitable questions from the test bank, and meanwhile the practical GPTPE was expanded to cover 4 disciplines with 7 questions, which was still so insufficient that no significant differences were observed between groups.

## Discussion

### GPs in Pudong new area

Pudong New Area is located in the east of Shanghai, covering an area of 1429.67 km^2^, 22.55% of the total area of the metropolitan city, and by 2015, the area had had a population of 5.47 million [32]. As revealed by the current study, there were 1264 GPs there, with the coverage of 2.25 and 2.31 per 10,000 residents in 2014 and 2015, respectively. As required by the Guidance of the State Council General Office on Promoting the Construction of Stratified Medical System, the nationwide staffing objective is to ensure 2/3 qualified GPs per 10,000 residents for primary care and first-contact services [33]. As reported in 2014, the national average numbered 1.27 GP per 10,000 residents [34], and accordingly Pudong New Area has been above the average.

To address the growing demand for the routine work of GPs at CHSC, however, the proportion should have at least 5 GPs per 10,000 residents in Shanghai [10]. In USA, UK, Canada, Australia, etc., every population of 2,000-3,000 can have a GP; in UK as a case in point, the proportion ranged from 6.1 in Northern Ireland to 8.2 per 10,000 population in Scotland in 2011 [35]. Definitely, it is imperative that further investigations be conducted on ensuring the standard and quality services on the part of GPs.

As evidenced by the current study, the GPs working in Type C and D of region were significantly older, with longer employment span and lower professional title than those working in Type A and B of region (*P* < 0.05), which suggested a relative inequality of quality GP resource in the different socioeconomic developing regions. According to the human capital theory, human capital with high quality can improve the output of medical services [36]. Compared with the urban areas, the existing problems such as insufficient number, under-qualified skill and frequent turnover were reported to be the bottleneck in improving the medical care system [37]. And the higher level of inequitable distribution of qualified health workers in those disadvantaged areas might have lower densities of the professionals [38]. Indisputably, there are growing needs for qualified and stable grass-roots health employees to deliver rural primary care; Pudong New Area is no exception. To meet the great challenge and achieve real fairness, the central government of China should keep an eye on the issue of “quality fairness,” directing further investigations on equity in quality.

Nowadays in China, most of the GPs working for CHSCs used to be specialist practitioners before job-transfer training; they are not so qualified due to their limited general medicine training [39, 40]. As evidenced by the scores of the theoretical examinations, the majority of the GPs was knowledgeable about such a discipline as internal medicine or gynecology, but did not have comprehensive medical knowledge as a qualified GP should possess. The shortage of qualified GPs cultivated by the “5 + 3” mode is a common phenomenon in China [41, 42], which is composed of 5 years of undergraduate medical education plus 3 years of GP standardization training. This is also true of Pudong New Area, where less than 10% of the GPs working at CHSCs were cultivated as required by the mode. Therefore, one of GPTPE’s strategic goals is to build qualified GPs to be real gatekeepers. The current study suggests that the first two-year evaluating results of developing GPTPE can provide valuable reference information for the actual construction of qualified GPs in China.

### Development of GPTPE Model

In theory, GPTPE is of profound importance in building qualified GPs in China. As evidenced by the data comparison between 2014 and 2015, efforts were made to improve Pudong-New-Area-based model of GPTPE in the aspects of discipline, content and management. However, there sure exist the issues of improvement and perfection in its further development so that the model of GPTPE can be institutionalized and normalized in a sustainable way.

In 2015, the theoretical item bank of GPTPE grew to possess 1040 questions specifically targeted at TCM GPs; thus the examination was separately scheduled for the TCM examinees. This change is not alone; in some other countries, the delivery of services requires differently skilled and different types of health professionals working in general practice [43]. In reality, as an important part of medical practice in China, TCM can be applied as the effective means of disease prevention and health-keeping behavior, deeply rooted in the Chinese residents who are willing to seek TCM for their health problems, as evidenced by some reports that the approach of TCM can meet the demand of the growing CHS [33, 44, 45]. In the current study, TCM GPs’ average score was still lower than that of WM GPs in 2015 theoretical examination (*P* < 0.05), which could be explained by their different professional competences and/or by their different testing competences due to the designing of the questions themselves. Such a phenomenon needs further investigations to verify.

Despite the expansion of the item banks of GPTPE from 2014 to 2015, the total average score was still lower than 60/100 in the theoretical and no differences were observed between different groups in the practical. This indicated that the item bank still remains to be desired, which was derived from the item bank of GP standardization training in the tertiary hospitals of Shanghai, and was improved by consulting experts. After the two-year implementation in Pudong New Area of Shanghai, it was found in the current study that the problem of feasibility and reliability still existed for the GPs working for CHSCs. According to the examinees’ feedbacks, the item banks of GPTPE were reported to be unsuitable for them.

In order to make the GPTPE model more conducive to qualified GP construction, further research is needed to explore ways to combine the evaluating scores with the on-going regional GP-based continuing medical education, performance appraisal and incentive mechanism. As to the social recognition and patient-physician harmony, there is still a need to explore appropriate and effective means to improve GPs’ real competence and promote CHS for their local contracted residents. On an administrative level, much needs to be done to take the advantage of the GPTPE model to inspire GPs’ enthusiasm and their work initiative, as well as to make full use of the washback effect on GPs’ academic and skillful improvement.

### Limitations

The current study was of a tentative research, which had much to be desired in evaluating the Pudong-New-Area-based GPs’ theoretical competence and practical performance. As one of assessments, GPTPE can be insufficient for a comprehensive evaluation; further studies need focus on exploring multiple dimensions of GPs’ evaluation. Additionally, the two-year data could not be sufficient enough to be an effective and valuable evaluation for intervention policies; therefore a bigger database based on a long-term observation is needed to better evaluate the newly developed model of GPTPE.

## Conclusions

As empirical evidence for the national establishment of GP evaluation mechanism as well as for the promotion of equity in the human resource for the delivery of primary health care across the country, the newly developed GPTPE in Pudong New Area is of profound importance in building qualified GPs in China. It can be concluded from the current study on the GPTPE to empirically evaluate GPs’ competence and performance, the resource of qualified GPs was not sufficient at CHSCs, which reflected the urgency of building qualified GPs to address the growing demand for CHS. As a preliminary study, it needs further research to explore ways of alliance for the existing CME, evaluating model and incentive mechanism in building qualified GPs.

## Abbreviations

CHFP: Commission of Health and Family Planning
CHS: Community health service
CHSC: Community health service center
CI: Confidence interval
CME: Continuing medical education
GP: General practitioner
GPTPE: GP’s Theoretical and Practical Examination
ME: Ministry of Education
MF: Ministry of Finance
MHRSS: Ministry of Human Resources and Social Security
MIAC: Medical Institutions Administration Center
NDRC: National Development and Reform Commission
NHFPC: National Health and Family Planning Commission
SCOPSR: State Commission Office of Public Sectors Reform
SD: Standard deviation
TCM: Traditional Chinese medicine
WM: Western medicine
